# Coordinated hospital-home care for kidney patients on hemodialysis from the perspective of nursing personnel^1^

**DOI:** 10.1590/0104-1169.0058.2546

**Published:** 2015

**Authors:** Luz María Tejada-Tayabas, Karla Lizbeth Partida-Ponce, Luis Eduardo Hernández-Ibarra

**Affiliations:** 2 PhD, Full Professor, Facultad de Enfermería, Universidad Autónoma de San Luis Potosí, San Luis Potosí, SLP, Mexico. Scholarship holder Consejo Nacional de Ciencia y Tecnología (CONACyT), Mexico; 3 Specialist in Nursing, RN, Clinica 50 Instituto Mexicano del Seguro Social, San Luis Potosí, SLP, Mexico. Scholarship holder Instituto Mexicano del Seguro Social, Mexico

**Keywords:** Hemodialysis, Kidney Disease, Health Services, Evaluation, Mexico

## Abstract

**OBJECTIVE:**

To examine, from the nursing perspective, the needs and challenges of coordinated hospital-home care for renal patients on hemodialysis.

**METHODS:**

A qualitative analysis was conducted with an ethnographic approach in a hemodialysis unit in San Luis Potosi, Mexico. Semistructured interviews were conducted with nine nurses, selected by purposeful sampling. Structured content analysis was used.

**RESULTS:**

Nurses recounted the needs and challenges involved in caring for renal patients. They also identified barriers that limit coordinated patient care in the hospital and the home, mainly the work overload at the hemodialysis unit and the lack of a systematic strategy for education and lifelong guidance to patients, their families and caregivers.

**CONCLUSIONS:**

This study shows the importance and necessity of establishing a strategy that goes beyond conventional guidance provided to caregivers of renal patients, integrating them into the multidisciplinary group of health professionals that provide care for these patients in the hospital to establish coordinated hospital-home care that increases therapeutic adherence, treatment substitution effectiveness and patient quality of life.

## Introduction

In Latin America, the prevalence of chronic kidney disease (CKD) has increased 6.8% annually over the past six years^(^ 1 ^)^. Mexico is one of the most affected countries, where more than 100,000 people have CKD, and the mortality rate is 10.5 per 100,000^(^ 2 ^)^. 

For people with CKD, one of the chosen replacement therapies for renal function is hemodialysis (HD), which is considered a highly invasive treatment and is one of the more costly chronic therapies in specialized care, as it involves high economic, physical and psychosocial costs for the patient and their family. CKD requires direct and continuous care of the patient at home and at the institution providing health care services. 

Nurses play a very significant role in the care of people who are admitted to the hospital for this disease, as they have close and continuous contact with the patient, and the greatest responsibility of care falls on these professionals. This onus is due to the specialized technical ability required for HD therapy and the need for continuous education and guidance to the patient and their caregivers to promote therapeutic follow-up in the home. Nurses then constitute the axis that brings together the series of actions that includes comprehensive care for renal patients on HD, which involves both the team of professionals and the informal caregivers of these patients. 

Various studies on the care of renal patients on HD have been reported. Studies with a quantitative approach mainly overlook analysis of quality of life and the needs of people subject to this replacement therapy^(^ 3 ^-^ 4 ^)^ to assess their functional capacity and physical activity requirements^(^ 5 ^)^. 

In contrast, qualitative studies analyze and interpret experiences of those with CKD receiving HD therapy to assess and understand their life experiences, meanings and impact of the disease on their lives^(^ 6 ^-^ 7 ^)^. Several studies have assessed the experiences of patients in particular on some specific symptoms of CKD and the effects of HD therapy, such as fatigue or pain^(^ 8 ^-^ 10 ^)^, along with prescriptions of medical treatment, for example fluid restriction^(^ 11 ^)^.

Some ethnographic studies have investigated the perceptions of the various actors involved in hospital care of patients on hemodialysis. For example, the study by Fujii^(^ 12 ^)^ describes factors that influence integration of care for patients with hemodialysis from the perspective of health personnel. The Allen study^(^ 13 ^)^ presents the perspective of patients regarding failure of care during their stay at the hospital for disease management.

Very few studies analyze patient perception regarding care practices in the home, such as the central venous catheter ^(^ 14 ^)^. Study publications in this region that inquire about the multiple related aspects of the requirements and experiences of care in the home were not identified, let alone studies that consider the continuous and coordinated hospital-home monitoring process of these patients. 

This study aimed to examine, from the perspective of nurses in a hemodialysis unit (HDU) in social security services, the needs and challenges of coordinated hospital-home care for comprehensive care of renal patients on HD. 

## Methods

This study presents results from a qualitative evaluation^(^ 15 ^-^ 16 ^)^ of care for renal patients on HD from the perspective of health personnel (nurses, doctors, psychologists, nutritionists, social workers, physiotherapists), the patients and their family. The research was conducted using an ethnographic approach^(^ 17 ^)^ in the Hemodialysis Unit (HDU) of a social security clinic in San Luis Potosí, Mexico, conducting interviews with nurses from January to May 2013. Six women and three men participated in this part of the study, totaling nine out of 15 professionals that work in the HDU on three different shifts who have an average age of 35 years of experience; two are nursing technicians, four registered nurses, and three nurse specialists in critical care, surgical nursing and nephrology, respectively. 

Participants were selected by convenience sampling considering voluntary participation in the study as the criterion; members of the nursing team who did not participate expressed their refusal as due to lacking time.

First contact with participants was established at the beginning of the study. At a face-to-face meeting, the significance of the research project was communicated to them as collaboration between the clinic and the institution that the principal investigator belongs to, along with the objectives of the study and the interest of the interviewers to obtain information for compliance. 

**Figure 1 -:**
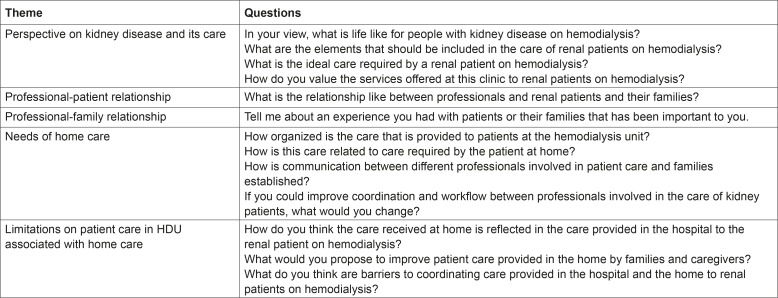
Interview Guide

Semi-structured interviews were conducted following an interview guide (Figure 1), (18 in total, two per participant, with an average length of 45 minutes) by the first two authors, LMT (PhD and researcher with extensive experience in qualitative methods, studies on chronic diseases and evaluation of health services) and KP (nurse with a Master's degree in Public Health) until thematic saturation was reached. Interviews were conducted individually in the clinic in a private place and were audio-recorded with consent of the informants and then transcribed in a word processor by KP. 

Participant observations were also made by KP, who was involved in daily activities of the HDU for care of the renal patients, with the purpose of knowing and taking into consideration the context in which hospital care is given and the characteristics of patients who come to receive care. Field notes, methodologies and analytics were prepared for records of field work and data analysis^(^ 18 ^)^. Participants were given a questionnaire to obtain personal data. 

**Figure 2 -:**
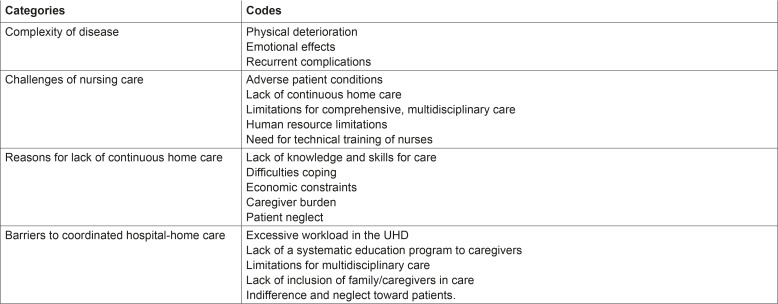
Categories and analysis codes

To analyze data, structured content analysis was conducted^(^ 19 ^)^, which allowed subjective interpretation of the content of texts through systematic steps of coding and identification of themes that enable following an inductive method to reach interpretation in a circular and permanent process. This method was developed consecutively with collection of information; three investigators participated in this process. The steps were as follows: 1) literal and systematic transcription of interviews; 2) each investigator performed a detailed reading of all transcripts and initial coding to identify significant themes that emerged from the text; 3) the various encodings were pooled to integrate a system of unique codes; and 4) the first author, with the same scheme, made a second coding of the transcripts, identifying consistencies and variability in the narratives, to place basic ideas shared among the informants and any differences in responses. The main categories derived from the analysis are presented in Figure 2. Results were discussed among investigators to reach consensus. The Atlas Ti 5.2 program (Thomas Muhr, University of Berlin, Germany) was used for analysis. Finally, results were presented to participating nursing personnel in the study and a few others who work in the HD unit to receive their feedback, which confirmed the findings^(^ 20 ^)^. 

The project was approved by the ethics and research committee of the institution in which the study was conducted. Those involved participated under informed consent expressed verbally. Ethical principles^(^ 21 ^)^ were addressed regarding autonomy, self-determination and guarantee of confidentiality of information. 

## Results

### Complexity of nursing care of renal patients

For interviewed nurses of the HDU, care of renal patients is challenging for several reasons: a) complexity of the disease and the way in which it is embraced by the patient and their family; b) responsibility of care mainly falls on nurses, although various physical and emotional ailments and the gradual deterioration of the patient merit comprehensive care that involves participation of a multidisciplinary team; and c) these patients require special care in the home so that therapeutic adherence is guaranteed - this type of care is not provided to the patient as required. 

Nursing staff participating in the study expressed that CKD and HD treatment constitute different conditions for the patient; that is, hemodialysis therapy becomes another ailment that is suffered by both the patient and their family, as it has adverse effects that must be offset by strict adherence to various additional therapeutic measures to the HD procedure. Thus, both CKD and HD generate a multitude of needs and problems in patients that most often cannot be satisfied by the patients themselves and cannot be resolved during their stay at the HDU; therefore, they require continuous home care.

As recounted by the nurses, a large portion of patients treated in the HDU suffer from more than one disease; additionally, deterioration is gradual and sometimes accelerated, which depends on age of the patient and therapeutic adherence at home. If deterioration of the patient is greater, their physical and emotional states are more complex, which clearly increases workload for nurses in the HDU and certainly warrants the intervention of other professionals to provide specialized quality care to the patient. Two nurses express these issues: 

*...there are patients who have been deteriorating, even some between 40 and 50 years. They are consumed, and it is not just the disease, hemodialysis wears them out a lot...* (Participant 1)

*...those who are younger fare better; older patients fight more because of the combined influence of hypertension, diabetes, kidney disease and many other factors, and a young patient can cope better. Therefore, it is necessary to receive complete care from a team of professionals. We as nurses cannot meet all their needs, care received in the home also influences a lot... *(Participant 4)

However, home care of renal patients generates complications and challenges the organization of family activities and family economics; the manner in which families cope with such complications to resolve them affects patient welfare. According to the nurses' narratives, it is very common for patients to discontinue HD treatment, sometimes from difficulties with transfer to the hospital due to their economic situation (mainly foreign patients) or because they do not have anyone to accompany them due to the difficulties involved for their caregivers to leave work or family commitments. As a result, when treatment stops, the physical condition of the patient worsens, and they often come to the hospital to seek care for emergencies. One of the nurses recounts this type of situation: 

*...it is common that many patients stop coming for some time or miss weekly [HD] sessions. When that happens, what makes them come back is complications from not undergoing hemodialysis. They start with pulmonary edema, and that is what forces them to return, but already with greater deterioration. This often occurs because it is difficult for the family to handle many situations they face with the patients. *(Participant 3)

## Need for continuity of care in the home

Participating nurses in this study recount some difficulties in providing care to their patients within the HDU due to lack of care for patients in the home. For example, the fact that patients do not follow their prescribed diets hinders the HD procedure. It is also common for patients to arrive in poor hygienic condition or even with dysfunctional (i.e., obstructed) vascular access or signs of infection. Additionally, it is very common that patients express depression and clear resistance to receiving treatment. 

In the home care context, these conditions reveal a lack of continuity and follow-up of attention that the patient receives in the hospital. Coordinating care provided to the patient in the hospital and the home is essential for hemodialysis therapy to fulfill its purpose and to be performed smoothly and to maintain patient wellness and quality of life. Some participants comment on these aspects: 

*... these patients are more susceptible to infections, they are informed about the care they should follow, but it does not make them aware, they are not given care that vascular access requires and often come with signs of infection... *(Participant 3) 

*... it is common to have problems because patients do not follow directions in their home, some say, "today I drank plenty of water, as it was time for hemodialysis". Some drink plenty of fluids and eat too much on the day scheduled for hemodialysis, this combined with regularly failing to follow the diet strictly...* (Participant 5) 

*... suddenly [care] gets complicated because we do not know how to treat them [the patients]. They have many needs in addition to physical and emotional ones. Patients commonly arrive irritable, angry and resistant to those who perform the procedure, and there are other patients who arrive crying, discouraged, depressed - the truth is sometimes we do not know how to help them... *(Participant 4)

According to accounts from nurses, patients newly admitted to the HDU and their families are given general guidance on home care and are even provided basic information leaflets; however, these measures are not sufficient to ensure continuity of hospital care in the patient's home.

## Barriers for coordinated hospital-home care

The main barriers expressed by the nurses for providing coordinated hospital-home care are: a) workload at the HDU as a result of the demand of service due to the lack of nurses and other professionals; b) limitations for a multidisciplinary team of professionals to be involved in the care of these patients, taking caregivers and family into consideration as part of the care process; c) lack of a systematic strategy for education and lifelong guidance to patients, their families and caregivers and; d) conditions of indifference and neglect some patients suffer from their family. 

From the perspective of nurses participating in the study, patient care in the HDU in which they work has greatly improved in recent years. However, it is impossible for the clinic and HDU to grow according to demand; the CKD problem continues to increase enormously, which creates difficulties for providing quality care to all users of the service. Clearly, the demand for care exceeds resource availability, mainly in the afternoon shift (Table 1). 

**Table 1 -:** Characteristics of the Hemodialysis Unit, Social Security Clinic, San Luis Potosí, Mexico, 2013

Characteristic	Morning shift	Afternoon shift	Accumulated work day	Total
Number of patients seen	72	43	12	127
Nurses	7	4	4	15
Other professionals				
Medical specialists	1	2	1	4
Social workers	1	-	-	1
Nutritionists	1	-	-	1
Psychologists	1	-	-	1
Physiotherapists	1	-	-	1
# of HD machines	15	15	15	15
Availability of material resources	Sufficient	Sufficient	Sufficient	
Availability of medicines	Sufficient	Insufficient	Insufficient	
Support services Laboratory	Sufficient	Insufficient	Insufficient	
Cabinet				

In this sense, nursing staff in the HDU are considered to have a work overload because this staff is responsible for the care and satisfaction of the multiple needs of each patient, all in the period of time in which the patient remains in the HDU. Thus, it is considered necessary to increase trained staff that will provide higher quality care and maintain close communication with the patients' families. This issue was expressed by a nurse: 

*The [HD] unit has grown to try to meet demand, although it is impossible because there are more and more patients. Before there were three machines, then 10, right now we have 15, before no more than 20 patients were seen per day, now we serve approximately 25 patients only counting the morning. *(Participant 1) 

*... the HDU should have sufficient nursing staff specialized in the area, a staff that has knowledge and skills to detect problems in these patients and to solve them according to our job... *(Participant 7)

Another important limitation that hinders development of coordinated hospital-home care is the lack of collaborative work between different health professionals (i.e., nephrologist, medical internist, vascular surgeon, psychologist, nutritionist, social worker, and physiotherapist, among others) who should be involved in the comprehensive care of renal patients. Principal among the limitations that prevent close coordination between professionals is the small number of medical specialists and other professionals to meet the large demand for care of these patients and others who come to the clinic. An example of this limitation is the fact that a specialist assesses patients on HD once or twice a year, which prevents closer monitoring and makes it almost impossible to involve families in patient care. A nurse expresses this problem: 

*...ideally one would work with a multidisciplinary team because care involves more than [the patient] coming to hemodialysis and that's it. The kidney patient requires care from different professionals, but here there are only two nephrologists with so much work that they see each patient once or twice a year. Also, there is only one vascular surgeon at the hospital, they give appointments with a delay of up to four or five months. So it is a problem, and if it is complicated for us to organize and coordinate, how can we do it with caregivers?... *(Participant 1) 

With regard to other professionals, such as the nutritionist, psychologist, physiotherapist and social worker, nurses recount that they have very little contact with patients; their activities are more focused on giving general guidance to the family, but without coordinating with the nursing team. Thus, these professionals can hardly keep track of therapeutic adherence of patients to HD therapy. Some participants allude to these issues as follows: 

*In the clinic, there is an area of psychology, another for nutrition, and it is assumed that we can send a patient to see psychology and that the nephrologist sends them to nutrition, but I think it has not worked out well, I do not know if it is because of the workload...* (Participant 4) 

*...in general, most patients would need physiotherapy support because most have physical limitations, many cannot even get off of the sofa. I believe that even if physiotherapy staff cannot give them rehabilitation, they could guide and teach the families on physical activity and movements that patients should practice at home...* (Participant 6) 

A major barrier for establishing coordinated care for these patients is the lack of a systematic approach that integrates all professionals and the informal caregivers. Additionally, it is common that some patients show signs of neglect from their families; many patients come to treatment alone. Therefore, it is difficult for nurses to have direct contact with the family, as recounted by a nurse: 

*...there are some patients who do not have a relative to accompany them and come alone, although they can walk, they are no longer able to walk alone. I explain something to them, and they do not understand. If there is no other person, a relative to whom to explain a situation that is presented to the patient, that too is complicated for us. We need permanent guidance activities for patients and their caregivers, but with so much work, how?...* (Participant 8)

## Discussion

The study findings show the complexity of therapeutic HD and nursing care in the hospital, which require, more than other areas of hospital care, continuity of care in the home of the patient. Moreover, the main barriers to coordinated care in the hospital and the patient's home are described. On the one hand, these barriers are associated with difficulties for the family to face the burden of the disease and its economic, emotional and social consequences. On the other hand, the availability of human resources, organization for care and excessive workload in the hospital setting hinder comprehensive patient care, including their caregivers, as these medical issues require specific training for them and close and continuous coordination and communication. 

These actors require a set of knowledge and specific skills to maintain strict therapeutic adherence in the home that involves drastic lifestyle changes and the performance of procedures for continuous care. Likewise, they require a support strategy that will give them mechanisms to cope with the burden of care. However, informal caregivers receive neither the training nor support needed, making it more complicated for them to take that responsibility and exercise their functions effectively. Several studies refer to the significance of this care and its relationship to quality of life for renal patients on hemodialysis^(^ 6 ^-^ 7 ^,^ 22 ^)^; some authors have reported the significance of active participation of the patient and their caregivers in the care process required in hemodialysis treatment, along with communication between patients, caregivers and health personnel^(^ 4 ^,^ 23 ^)^. Other studies have investigated the perspective of professionals and patients on hospital care^(^ 12 ^,^ 13 ^)^, and findings are consistent with this study in regards to limitations of human resources, the long waiting times and difficulties in coordinating the professionals involved in the care of these patients.

Moreover, the need for guidance, education and constant support from the health team for the patient and the family so that they can more easily accept and face radical lifestyle changes has already been documented^(^ 3 ^,^ 5 ^)^. However, there is no evidence of the implications and barriers to establishing coordinated hospital-home care that involves collaborative, systematic work between health professionals, caregivers/relatives and patients. 

This study shows the lack of a systematic strategy in the institution in which the study was conducted, a strategy involving coordination of a multidisciplinary team and relatives or informal caregivers of these patients to provide comprehensive care to the patient. Such a strategy, in addition to including education and sufficient training for families to care for the patient in the home, should offer them support alternatives to face both their relative's disease and their care requirements so as to achieve more effective and personalized care that considers particular conditions of each patient and their family. Coordinated hospital-home care would increase patient quality of life^(^ 24 ^)^ and the level of hope that they can develop and would also consider and provide support to the caregiver^(^ 25 ^)^. 

Moreover, the increase in patients requiring kidney replacement therapy with HD is challenging due to the increased activity and use of human and material resources for various additional hospital services, such as emergency consultations, surgery, internal medicine and intensive care. The various complications of both the disease and its treatment are the fundamental causes of frequent hospitalizations, low patient quality of life and excessive caregiver burden. Coordinated hospital-home care would increase patient quality of life; therefore, it might be possible to decrease demand for hospital services and reduce the waiting times from different hospital departments attending complications. 

The results from this study are unique to the institution in which they took place, though some of the findings could be transferred to other hospitals that provide HD care. It is necessary to conduct the study in other care institutions, involving the perspective of patients and their caregivers and the rest of the professional team. A study that addresses this diversity of perspectives is in progress and will certainly enrich the possibilities of presenting a feasible proposal for educating caregivers of renal patients with a coordinated strategy. 

## Conclusion

The results of this study highlight the significance of establishing coordination between health professionals and caregivers to maintain therapeutic compliance in the home and to establish a multidisciplinary support network for both actors, which will facilitate the nursing staff in caring for the patient in the HD room and could reduce demand for hospital services from complications associated with the lack of home care. Thus far, actions to educate and guide caregivers of renal patients do not consider establishing a coordinated strategy that integrates caregivers in a multidisciplinary team and provides them tools in terms of knowledge, skills and strategies to face the difficult burden of home care for these patients. 
